# Effect of Animal Age at Slaughter on the Muscle Fibres of *Longissimus thoracis* and Meat Quality of Fresh Loin from Iberian × Duroc Crossbred Pig under Two Production Systems

**DOI:** 10.3390/ani11072143

**Published:** 2021-07-20

**Authors:** Alberto Ortiz, David Tejerina, Susana García-Torres, Elena González, Javier Francisco Morcillo, Ana Isabel Mayoral

**Affiliations:** 1 Meat Quality Area, Center of Scientific and Technological Research of Extremadura (CICYTEX-La Orden), Junta de Extremadura, Ctra, A-V, Km372, 06187 Guadajira, Spain; alberto.ortiz@juntaex.es (A.O.); Susana.garciat@juntaex.es (S.G.-T.); 2 Animal Production, Escuela de Ingenierías Agrarias, Research Institute of Agricultural Resources (INURA), University of Extremadura, Avda. Adolfo Suarez s/n, 06007 Badajoz, Spain; malena@unex.es; 3 Department of Anatomy, Cell biology and Zoology, Faculty of Sciences, University of Extremadura, Av. de Elvas s/n, 06006 Badajoz, Spain; morcillo@unex.es; 4 Department of Animal Medicine, Faculty of Veterinary, University of Extremadura, Av. Universidad s/n, 10071 Cáceres, Spain; amayoral@unex.es

**Keywords:** muscle fibre characteristics, age at slaughter, Iberian crossbred pigs, *Longissimusthoracis*, *Montanera*

## Abstract

**Simple Summary:**

This paper is in attempt to assess the impact of the production system *Montanera* and intensive, and the age at slaughter in each of them, on the muscle fibres of *Longissimus thoracis* (LT) and meat quality of fresh loins from Iberian pigs. The age at slaughter affected the fibre size of *Montanera* LT muscles, while the production system influenced the fibre type I population and type IIB fibre size. The age at slaughter affected the fresh loin quality trait depending on the production system; *Montanera* and intensive fresh loins from older animals yielded higher myoglobin and redness values. Additionally, intensive fresh loins derived from older animals yielded the lowest intramuscular fat and the highest cook loss and shear force in the Warner-Braztler shear force test. These above-mentioned parameters differed due to production systems, *Montanera* fresh loins less fat-infiltrated and tough but with higher redness values. The results of the present study contribute to shedding light regarding how the production-related factor animal slaughter age may influence the muscle fibre population, size and quality traits of fresh meat.

**Abstract:**

Two production systems and several ages at slaughter were used: 12, 14 and 16 months for outdoor rearing (with the final finishing phase in the *Montanera* system, in which fed was based on natural resources, mainly acorns and grass) and 8, 10 and 12 months for animals reared indoors (intensive system: with feed based on commercial fodder) to evaluate their effect on the muscle fibre population and size of the *Longissimus thoracis*, (LT) muscle, as well as fresh loin quality traits. Animals that were older at slaughter revealed increased fibre sizes of the LT muscles in the pigs reared in the *Montanera* system. The LT muscles of the animals reared in intensive systems had a lower percentage of type I fibres and higher size of type IIB than those reared in the *Montanera* system. The approximate composition and instrumental colour of *Montanera* fresh loins were affected by the animal slaughter age. In the case of the intensive system, the effect of animal slaughter age had an impact on the approximate composition, instrumental colour, water loss and textural properties. Therefore, different ages at slaughter of Iberian pigs showed variations in some quality parameters in the fresh loins in both the *Montanera* and Intensive systems, thus proving to be a factor of variability and homogeneity of the Iberian products. The meat from Iberian pigs reared in an intensive system and slaughtered at a younger age proved to be more tender. The production system affected all the above quality traits, with the exception of water loss.

## 1. Introduction

Meat and meat products derived from Iberian pigs (autochthonous breed pig reared in the Southwest of Spain) are highly valued and accepted products at national and international markets [1], mainly due to their high quality and organoleptic characteristics.

It is well-known that both the production system and breeding have an influence on the quality of meat and meat products. The traditional Iberian pigs finishing system is linked to the *dehesas* (*Quercus* sp. open woodlands that are found in the Southwest of the Iberian Peninsula), in which the animals are fed exclusively on acorns and grass. Animals finished in *dehesas* result in high-quality products. However, the geographical limitations of *dehesas*, as well as the natural resources seasonality (November–March), have led to a diversification in the way Iberian pigs are produced in order to face the growing demand of Iberian products [2]. Thus, the intensification of the management system and a feeding program based on commercial feedstuffs has become an alternative to the traditional *Montanera* production system. In addition, the requirements to keep production costs low have led to the spread of Iberian × Duroc (IB × D) pig breeding programmes across farms [3], given their better productive parameters compared to purebred animals [4]. This results in crossbred animals being able to reach weights similar to those of purebred Iberian pigs at a younger age and, therefore, reduces the production cycle. Nevertheless, animal age at slaughter is regulated by the current legislative framework—the Spanish Iberian Quality Standard (SIQS) [5]—that is linked to the production system, despite the growth rate of the animals used (Iberian purebred or crossed with Duroc). Specifically, for animals being reared in the *Montanera* system, the minimum age at which they must be slaughtered is 14 months, while, for animals in intensive systems, this is 10 months. Therefore, animal age at slaughter may generate disagreement between the requirements of the current regulatory framework and the interests of farmers, who typically use this breed to increase productivity and reduce costs [3].

On the other hand, the shortening of the production period may bring about alterations in the quality of the meat [6,7], which could be associated with *post-mortem* changes in the conversion of muscle to meat and fibre-type composition [8,9,10]. Previous studies have concluded age-related changes on approximate compositions. More in detail, Lorenzo, Fernández, Iglesias, Carril, Rodríguez and Franco [11] observed higher values of dry matter with animal age in meat from Celtic breed pigs, whilst Bosch, Tor, Reixach and Estany [12] observed that intramuscular fat increased with animal age in fresh loins belonging to Duroc breed pigs. Additionally, the myoglobin content of the muscle (and, in consequence, the meat instrumental colour), the insoluble collagen (affecting the textural properties of the meat) and the water losses might be related to the lifetimes of animals, as reported by Mayoral, Dorado, Guillén, Robina, Vivo, Vázquez and Ruiz [13] for purebred Iberian pigs. It is therefore important to raise awareness of the changes that animal age at slaughter may cause on muscle fibre characteristics and their repercussions on the meat quality of products.

The fibre type of classification is mainly based on the contractile and metabolic performance characteristics. Four major types of muscle fibres are found in the skeletal muscle of adult animals: (1) slow-twitch oxidative or type I, (2) fast-twitch oxido-glycolytic or type IIA, (3) fast-twitch glycolytic (IIB) and (4) fast-twitch oxidative (IIX) [14]. Type I fibres have a greater oxidative capacity to support sustained muscle contractions, whereas type IIB fibres are predominantly glycolytic fibres that utilise the rapid conversion of glycogen for short bursts of energy. The IIA and IIX types of fibres are between type I and IIB fibres. The sizes and proportions of the different muscle fibres can vary according to various production factors, such as breed [15], growth performance [16], physical exercise associated to free-range rearing [17] or diet [18], and so, their impact on the quality parameters must be taken into account. The scientific literature has addressed the influence of the genetic background (Iberian purebred vs. Iberian × Duroc crossbred), management and feeding conditions on the muscle fibres of various muscles that are present in ham [19]. However, to our knowledge, there are no age-related changes in the Iberian × Duroc crossbreed, despite this breed catching the interest of all the players of the Iberian pig industry.

There is a need to generate knowledge about the extent to which fibre types and sizes might be impacted by animal age at slaughter in the various production systems, considering the age ranges that are currently used and are suitable in each production system. The variations in fibre compositions may help understand the variations of some of the properties relating to meat quality and technological aptitude, such as colour, water loss or texture, and their importance in the improvement and control of meat quality without reducing the advantages that the fast growth performance of the Iberian × Duroc crossed animals provide.

Within this framework, the purpose of the current study was to evaluate the impact of animal age at time of slaughter of two production systems and the effects of the production systems themselves on the muscle fibre characteristics and approximate composition, water losses, instrumental colour and texture properties of the meat.

## 2. Materials and Methods

### 2.1. Animals and Experimental Design

A total of 48 Iberian Retinto (Valdesequera line, Junta de Extremadura, Badajoz, Spain) crossed with Duroc (IB × D) (50:50) animals were used. Animals were divided into two production systems: *Montanera* (*n* = 24) and intensive (*n* = 24). Each production system included animal batches that were slaughtered at three different ages, with 8 pigs per batch. For this purpose, the birth date of the animals within each batch was successive, and there was a timeframe of 2 months between them.

The males (4 per animal slaughter age batch, with a total of 24 animals) were surgically castrated following Spanish regulations [20]. Females (4 per animal slaughter age batch, with a total of 24 animals) were immunologically castrated by means of a vaccination against GnRF, which consisted of the application of three doses of 2 mL of IMPROVAC^®^ (Zoetis, Madison, NJ, USA) subcutaneously at their 20th, 24th and 40th weeks of life, with the exception of animals that were to be slaughtered at 8 months old reared in the intensive system that did not receive the third dose of the vaccine, given that they were slaughtered before their 40th week of life. The animals were reared in compliance with the European Union regulation [21] for minimum standards for the protection of pigs, which is transposed into the national legislation for the care and handling of animals by Royal Decree 1392/2012 [22] and for pigs reared in extensive systems by Royal Decree 1221/2009 [20]. Given that the experimental procedures to which the animals were subjected were considered standard husbandry practices, the present study was not subject for consideration in regards to the ethical and welfare aspects by the Animal Care & Ethics Committee (ACEC).

### 2.2. Animals Management under the Montanera System

**Figure 1:**
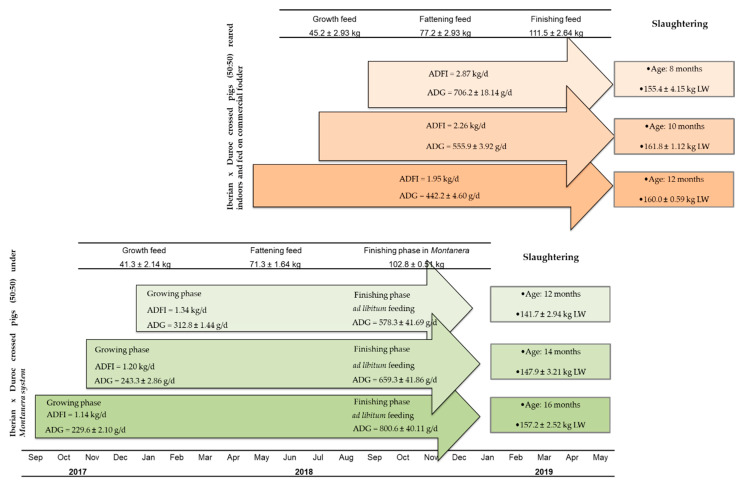
Experimental design of the animal production systems and batches according to slaughter age. Data expressed as arithmetic mean ± standard error. Animal average birth date: 2 September 2018, 4 July 2018 and 5 May 2018 for animals under an intensive production system and slaughtered at 8, 10 and 12 months, respectively, and 7 January 2018, 6 November 2017 and 4 September 2017 for animals under the *Montanera* production system and slaughtered at 12, 14 and 16 months, respectively. ADFI, average daily feed intake; ADG, average daily gain; LW, live weight.

In the *Montanera* production system, the three batches of IB × D pigs used were slaughtered at the age of 12, 14 and 16 months old. Prior to the finishing phase in *Montanera* (growing phase), the animals were managed under feeding restrictions in order to have them start the *Montanera* stage at different ages but similar live weights (LW). The animals were housed in open-air pens by batches, and their daily feed was provided individually by placing each pig in an individual pen where they received an amount of feed adjusted to their needs: 1.34, 1.20 and 1.14 kg/day of average daily feed intake (ADFI) for IB × D animals that were slaughtered at 12, 14 and 16 months of age, respectively. Thus, the animals yielded an average daily gain (ADG) during the growing phase of 312.8 ± 1.44, 243.3 ± 2.86 and 229.6 ± 2.10, respectively. The nutritional characteristics of the feed supplied—commercial feeds during the growing phase according to animal LW—are shown in Table 1. Subsequently, animals started the last finishing phase in *Montanera* at similar LW (103.3 ± 0.50, 102.9 ± 0.29 and 102.1 ± 0.66kg; mean ± standard error) but different ages (10, 12 and 14, respectively). During this finishing phase, animals were free-range-reared collectively at the Valdesequera *dehesa* farm, Junta de Extremadura, Badajoz, Spain from November 2018 to January 2019. The length of *Montanera* was 67 days, and the stocking rate was 0.60 pigs per hectare. During this period, animals had access to an ad libitum feed of acorns from *Quercus ilex* and grass (Table 1) and had free access to water. The ADG of the various animal batches during the finishing phase were 578.3 ± 41.69, 659.3 ± 41.86 and 800.6 ± 40.11 g/day for IB × D animals that were slaughtered at 12, 14 and 16 months of age, respectively. The animals were reared in the *Montanera* system until they reached the age established for slaughter (12, 14 and 16 months, with an average LW of 141.7 ± 2.94, 147.9 ± 3.21 and 157.2 ± 2.52 kg, respectively) (Figure 1).

### 2.3. Animals Management in Intensive System

In the intensive system, the ages at slaughter for the various animal batches were 8, 10 and 12 months old. In terms of the production system conditions, animals were reared in an intensive system, with a minimum living space of 2 m^2^ per animal. The animals that were slaughtered at 10 and 12 months of age were subjected to feeding restrictions, and their daily feed was provided individually by placing each pig in an individual pen, whilst the animals to be slaughtered at 8 months old were fed ad libitum. Therefore, the ADFI, according to the animal batches, were 2.87, 2.26 and 1.95 kg/day for IB × D animals slaughtered at 8, 10 and 12 months. This allowed for slaughter of the animals to take place simultaneously while they had similar live weights (155.4 ± 4.15, 161.8 ± 1.12 and 160.0 ± 0.59, respectively) (Figure 1). The nutritional characteristics of the commercial feeds supplied according to the animal live weights are shown in Table 1.

The nutrient requirements of the pigs were calculated according to the Spanish Foundation for the Development of Nutrition [23]. Feed consumption was monitored every three weeks by means of a weight control in order to adjust the weight increases to a previously defined theoretical growth curve, so that, in the case of the *Montanera* animals, the three batches could reach a similar weight at the beginning of the *Montanera* and, in the case of the intensive system, reach a similar slaughter weight regardless of the ages of the animals.

### 2.4. Slaughtering

After reaching the age established for slaughter, the animals were transported to a commercial slaughterhouse (Mafrivisa, Castuera, 6420, Spain) in compliance with the European Union regulation on the protection of animals during transport and related operations [24]. Subsequently, the animals were offloaded from the trucks and provided with water but no feed. Then, they spent less than 24 h in lairage prior to slaughter, which was carried out by exsanguination with previous carbon dioxide stunning, in compliance with the European Rules for the protection of animals during operations at the time of slaughter [25].

### 2.5. Sampling of Muscle Fibres

Samples were taken from the *Longissimus thoracis* muscle placed on the left side of the carcass. Specifically, they were collected from the area of the muscle located between the 5th and 6th ribs within 1 h after slaughter. The samples were cut perpendicular to the direction of the muscle fibres into 0.5 cm^3^ cubes: 1 cm × 1 cm × 0.5 cm (width × length × thickness). Immediately, samples were frozen in isopentane that was previously chilled in liquid nitrogen (−196 °C) [26]. Samples were stored at −80 °C until further analysis.

### 2.6. Meat Sampling

The carcass quartering was carried out in the short term after slaughtering (4 h *post-mortem*), i.e., hot quartering [27]. Subsequently, the whole *Longissimus thoracis* (LT) muscles were removed from the left side of the carcass and subsequently chilled at 4 °C for 24 h to be later used to evaluate the meat quality traits. For the meat quality analysis, the LT muscles were filleted from the cranial to the caudal area. Thus, the first steak (2 cm thick) was assigned to determine the instrumental colour and myoglobin content, water-holding capacity (WHC) and approximate composition, dry matter (DM) and intramuscular fat (IMF). The following two steaks (3.5 cm thick) were assigned to undergo a texture analysis and to determine the cooking loss (CL), respectively.

### 2.7. Methods

#### 2.7.1. Muscle Fibre Histochemistry and Morphometry Analysis

Serial cross-sections were taken at 9 μm in a cryostat (CM 1900 LEICA, Instrument GmbH, Germany) at −20 °C and placed on glass slides for the histochemistry analysis. These serial sections were stained for myofibrillar adenosine triphosphatase (mATPase) following the acid (pH 4.3, pH 4.55 and pH 4.6) preincubations [28]. The optimum pHs of the preincubation solutions were searched carefully to visually distinguish at least three levels (light, medium and dark) of staining intensities. The histochemical activity of nicotinamide adenine dinucleotide tetrazolium reductase (NADH-TR), an enzyme frequently used as a marker for the oxidative capacity of myofibers, was also qualitatively estimated on 9-μm-thick sections [28]. Histochemical serial sections were visualised with a Nikon H550S microscope (Nikon, Metrology Europe NV, Leuven, Belgium) and image analyser software NIS-Elements Br 2.30. All sections were carefully surveyed to find two regions that were free of artefacts that contained between 200 and 250 fibres. With the use of the mATPase staining stage after acid preincubation and the NADH-TR staining stage, muscle fibres were identified. The histochemical fibre types were classified into four major types (I, IIA, IIB and IIX). Once each individual fibre was identified, the minor diameter was determined. 

#### 2.7.2. pH Measurements

Muscle pH was measured at 45 min (pH_45_) *post-mortem* on the LT muscle from the left side of the carcass in the slaughterhouse and 24 h (pH_24_) *post-mortem* on the removed muscle in the meat laboratory. The pH was measured using a penetration electrode coupled with a temperature probe (Crison pH meter mod. 507, Crison Instruments, Alella, Barcelona, Spain). 

#### 2.7.3. Instrumental Colour and Myoglobin Content

Instrumental colour measurements were taken following the recommendations for colour determination of the American Meat Science Association [29]. A Minolta CR-400 colorimeter (Minolta Camera, Osaka, Japan) with illuminant D65, a 0° standard observer and a 2.5-cm port/viewing area was used. The colour coordinates determined were: L* (lightness), a* (redness) and b* (yellowness) and expressed as CIELab units (dimensionless). Additionally, the saturation index or chroma (C*), defined as $C*=\frac{\left({a*}^{2}+{b*}^{2}\right)}{2},$ and hue angle (H°), defined as $H{}^{\circ}=arctg=\frac{b*}{a*},$ were calculated. The measurements were repeated at five randomly selected sites on the exposed surface of each meat sample and later averaged.

The method of Hornsey [30] was applied to determine the meat myoglobin content (Mgb), and the results were expressed as the mg of myoglobin/g of meat.

#### 2.7.4. Approximate Composition

Dry matter was determined according to the AOAC method [31]. For this purpose, the samples were dried at 105 °C for 24 h until a constant weight was reached, and the results were expressed in g/100 g meat.

The intramuscular fat content was carried out following the methods used by Folch, Lees and Sloane-Stanley [32] by gravimetric measurements of the extracted fat weight, using chloroform/methanol (2:1, *v/v*) for the extraction.

#### 2.7.5. Water Holding Capacity

The water-holding capacity (WHC) was evaluated following the method proposed by Irie and Swatland [33]. This method measured the water released from the sample to dried filtering paper after the application of a centrifugal force (3000 rpm for 3 min). The results were calculated by the gravimetric difference and expressed as g of water released/100 g of meat.

#### 2.7.6. Cooking Loss

The fresh steaks were weighed and packed under vacuum conditions in nylon/polyethylene bags and cooked by immersion in a water bath preheated at 80 °C with controlled temperature until the steak reached an internal temperature of 75 °C [34]. Cooked samples were left to cool under tap water for 30 min in order to prevent further cooking, and then chilled overnight at 4 °C. The difference in weight before and after cooking was used to calculate the CL, and the results were expressed as water loss g/100 g of muscle.

#### 2.7.7. Instrumental Texture

The texture was instrumentally evaluated on cooked samples following the procedure described above. The texture analysis was performed using a TA-XT 2i Texture Analyser of Aname (Stable Micro Systems Ltd., Surrey, UK) texturometer. The instrumental determinations were repeated 8 times per sample, and the results were data averaged. Two texture analyses were carried out: A Texture Profile Analysis (TPA) and a Warner-Bratzler (WBSF) test.

- Texture Profile Analysis test

For the TPA test, the cooked samples were cut into uniform cubes of approximately 1 cm^3^ and were axially compressed to 20% of their original height using a probe with a 20-mm diameter flat plunger (P/20) (Stable Micro Systems Ltd., Surrey, UK) connected to a load cell of 25 kg at a test speed of 2 mm/s. The samples were compressed into two cycle sequences, according to the recommendations for analysing food textures provided by Bourne [35]. The TPA test with 20% of compression was used to determine the contribution of the myofibrillar structures, without the intervention of connective tissue, on the meat texture. The textural parameters obtained from the force–deformation curves were: hardness (N/cm^2^), springiness (cm), cohesiveness (dimensionless), gumminess (N·cm·s^2^), chewiness (N·cm·s^2^) and resilience (dimensionless).

- Warner-Bratzler Shear Force test

For the purposes of the WBSF test, the cooked samples were cut into 15 × 30 × 5 mm^3^ (width × length × thickness) pieces and cut in a perpendicular direction to the muscle fibres using a Warner-Bratzler blade (HDP/BS) in order to determine the maximum shear force (N/cm^2^). The results were expressed as WBSF (N/cm^2^).

#### 2.7.8. Statistical analysis

The statistical analysis was carried out using SPSS version 20.0 (IBM, Armonk, NY, USA). One-way ANOVA tests were carried out to study the effect of age at slaughter in each production system independently and to evaluate the effect of the production system on the muscle fibre population and size, pH assessment, proximal composition, instrumental colour and myoglobin content, water-holding capacity, cooking loss and texture characteristics. Thus, the model used was as follows:

Y_ij_ = µ + ST_i_ + e_i(j)_, (1)

where Y_ij_ is the variable considered; µ is the mean value; ST_i_ is the effect of slaughter age (i = 1:8, i = 2:10 and i = 3:12 months for intensive and i = 1:12, i = 2:14 and i = 3:16 months for the *Montanera* production system, respectively) or production system (i = intensive and i = 2: *Montanera*) and e_i(j)_ is the residual error.

The data are presented as the arithmetic mean ± standard error for each age group in each production system and between the production systems, respectively. Statistical significance was assessed according to Tukey’s HSD test, and the level of significance was set at *p* = 0.05. 

## 3. Results

No differences were found between the fibre proportions of the LT muscle on account of the animal age at slaughter in animals reared in either the *Montanera* (Table 2) or intensive system (Table 3) (*p* > 0.05). 

As far as fibre diameters are concerned, differences were identified on account of the age at slaughter on the LT muscles of animals reared in the *Montanera* system (Table 2) (*p* ≤ 0.05) but not on those of animals reared in the intensive system (*p* > 0.05) (Table 3). 

All the fibres of LT muscles from animals slaughtered at the oldest age, i.e., 16 months old, reached a greater diameter in comparison to the muscles from the animals that were slaughtered at 12 and 14 months of age (*p* ≤ 0.05). In the specific case of type I fibres, an intermediate diameter was identified in muscles from animals slaughtered at 14 months of age with respect to those from animals slaughtered at 12 and 16 months of age (*p* = 0.017). Thus, the diameters of type I muscle fibres from animals reared in *Montanera* increased with the age at slaughter (*p* = 0.017). This diameter was the largest at 16 months, intermediate at 14 months and the smallest at 12 months of age at slaughter (98.38, 89.65 and 82.28 µm, respectively). The highest value of the muscle fibre minor diameter was identified in animals that were slaughtered at 16 months of age: type IIA (96.31 µm; *p* = 0.001), type IIB (100.20 µm; *p* = 0.002) and type IIX (96.17 µm; *p* = 0.000).

The results of the muscle fibre type population and size according to the production system are shown in Table 4. Type I muscle fibre was predominant in muscles from animals reared in the *Montanera* system (*p* = 0.026), with 24.16% vs. 18.85%, respectively. Animals being reared in an intensive system did not have their muscles prepared for time-sustained exercising, and it was expected for them to have less type I muscle fibres. On the other hand, the production system only affected the size of type IIB glycolytic fibres, yielding higher values in the muscles from animals reared indoors than those from animals reared in *Montanera* (103.42 vs. 88.91 µm of the minor diameter, respectively) (*p* = 0.010).

**Figure 2:**
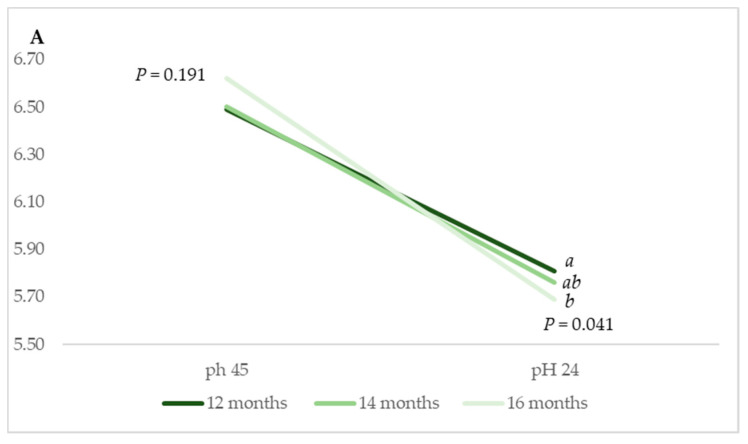

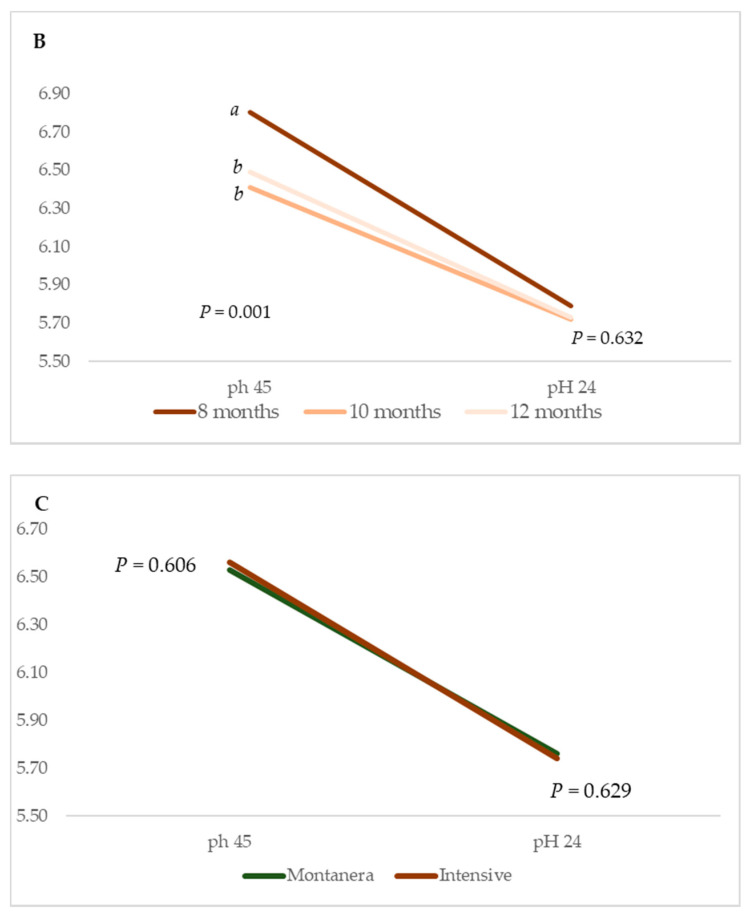
Arithmetic mean pH values measured in m. *Longissimus thoracis* from Iberian × Duroc crossed animals at 45 min and 24 h after slaughter according to the age at slaughter of the animals in each production system: *Montanera* at 12, 14 and 16 months (**A**) and intensive at 8, 10 and 12 months (**B**) and the production system itself (*Montanera* vs. Intensive) (**C**). Different letters indicate differences due to animal slaughter age within each production system for *p* = 0.05, according to Tukey´s HSD test.

Some differences were identified in terms of pH_24_ (*p* = 0.041) between muscles from animals reared in *Montanera* on account of the animal age at slaughter (Figure 2A). Muscles from animals that were slaughtered at 12 months of age yielded the highest value, i.e., 5.81, in comparison to 5.76 and 5.69 for LT muscles from animals slaughtered at 14 and 16 months of age, respectively. Additionally, the declining pH rate was more pronounced in LT muscles from animals slaughtered at 16 months of age compared to those of muscles from animals slaughtered at 12 and 14 months old.

With regards to the animals being reared in intensive systems, differences in the pH were identified 45 min after slaughter. The youngest animals yielded the highest pH value at this stage, i.e., 6.80 vs. 6.41 and 6.49, respectively (*p* = 0.001) (Figure 2B). In this case, the declining pH rate was more pronounced in LT muscles from the youngest animals (8 months). 

In contrast, no differences were identified in the pH measurements at 45 min or 24 h later on account of the production system (*p* > 0.05) (Figure 2C).

Table 5, Table 6 and Table 7, respectively, represent the way meat quality traits are affected by animal age at slaughter both in the *Montanera* and the intensive production systems, as well as the production system itself.

With regards to the *Montanera* production system (Table 5), the data revealed differences found in the DM and myoglobin contents of fresh loins on account of animal age at slaughter (*p* = 0.000). Thus, loins from animals that were slaughtered at 16 months of age yielded higher DM and myoglobin contents than loins from animals slaughtered at 12 and 14 months of age. A similar pattern was identified in terms of redness, with loins from animals that were slaughtered at 14 and 16 months of age yielding higher values than those slaughtered at 12 months of age (*p* = 0.025). No differences on account of animal age at slaughter were identified for water loss and the textural properties (*p* > 0.05).

Some of the meat quality traits evaluated also varied on account of animal age at slaughter when the animals were reared indoors (Table 6). The IMF content of the fresh loins from animals slaughtered at 12 months of age showed the lowest value (4.65%) with respect to 6.87% and 6.65% yielded by the fresh loins from animals slaughtered at younger ages (8 and 10 months, respectively) (*p* = 0.048). The myoglobin content followed a trend similar to that of fresh loins from animals reared in the *Montanera* system, with the values increasing as the animals were older at slaughter (*p* = 0.033). In contrast, no differences were identified in DM on account of the age at slaughter (*p* = 0.436). 

Water loss only showed significant differences in terms of cooking loss, and it increased with the age at slaughter, with the highest value being identified in fresh loins from animals slaughtered at 12 months of age (*p* = 0.002). As far as instrumental colour is concerned, the animal age at slaughter had a significant effect on meat redness. Therefore, higher a* values were identified in loins from animals slaughtered at the oldest age (12 months old) (*p* = 0.028). In terms of textural properties, differences in WBSF were identified, with fresh loins from animals slaughtered at 12 months of age yielding the highest values in comparison to animals slaughtered at younger ages (*p* = 0.014).

The meat quality traits of fresh loins were also affected by the production system in which the animals were reared (Table 7). With regards to the approximate composition, fresh loins from animals reared in the *Montanera* system yielded lower IMFs (*p* = 0.000) and higher myoglobin contents (*p* = 0.014) than animals reared in the intensive system. As far as instrumental colour was concerned, the production system had a significant effect on the lightness, redness and yellowness. Thus, the L* value was higher in fresh loins from animals reared in the intensive system (51.05) (*p* = 0.000), whilst the a* (*p* = 0.000) and b* (*p* = 0.043) coordinates were higher in fresh loins from animals reared in the *Montanera* system.

With regard to the textural properties, fresh loins from animals reared in the intensive system yielded lower values of maximum shear force (WBSF) (*p* = 0.035) but higher hardness (*p* = 0.000) and chewiness (*p* = 0.000) values than those from animals under the *Montanera* system.

## 4. Discussion

A lower proportion of type I fibres in the LT muscles of animals reared in intensive conditions compared to animals reared in the *Montanera* system (Table 4) was expected. Previous scientific literature agrees on the increase of the oxidative capacity of the muscles as the animal exercises [9,10]. Animals reared in the *Montanera* system were subjected to sustained exercise for longer periods of time, thus developing more resistance to fatigue and requiring more muscle energy. Thus, the higher type I fibres (slow-twitch oxidation) would be associated with a large oxidative ability to support sustained muscle contractions [9]. This supports the previous literature on the influence of the muscle function on the fibre-type population [10,36].

The lack of differences in the fibre-type populations on account of animal age at slaughter in any of the systems under study (Table 2 and Table 3) might be explained, because the most relevant changes occurring during the growth phase of the animals in terms of the fibrillary structure are associated with hypertrophy, this is an increase in size of the fibres [37]. In fact, significant differences were identified in the minor diameter measurements in all types of fibres of the LT muscle on account of age at slaughter of the animals reared in the *Montanera* system (Table 2). On the other hand, we must take into account that the animals were subjected to feed restrictions during their growing phase in order to be slaughtered at different ages with similar weights. This feed restriction could have impacted the size of the fibres [38]. Thus, Lefaucher and Ecolan [38] reported an increase in the fibrillary sizes of the *Longissimus thoracis* and *lumborum* and *Tibialis cranialis* muscles due to feed restrictions in Large White breed pigs with 100 Kg of LW. However, similar to the results obtained in this study, Lefaucher and Ecolan [38] did not identify variations in the fibre-type populations based on feed restrictions. 

The differences in size of the type IIB fibres on account of the production system (Table 4) could relate to feed type. Specifically, the highest average value identified in LT muscles from animals reared indoors would be explained by the higher intake of proteins that are contained in commercial fodders compared to the proteins provided by acorns and pasture in animals reared in *Montanera* (Table 1), as has been previously reported in other pig breeds [39]. In this respect, Andrés, Ruiz, Ventanas, Tejeda and Mayoral [10] also found type I fibres with larger cross-sectional diameters in the *Biceps femoris* muscle from animals reared in the intensive system compared to those reared from *Montanera*.

With regards to the association between meat quality traits and muscle fibre characteristics, although the muscle pH and the rate of *post-mortem* (p.m.) pH decline have been previously reported to depend on the fibre-type composition, the findings of our study did not prove this. Some previous studies agree on the increase of the rate and extent of the p.m. pH decline as the proportion of fast-twitch glycolytic fibres increase in porcine *Longissimus thoracis* and *lumborum* muscle [16,40,41]. However, despite that this study proved there were pH_24_ (Figure 2A) differences in the meat from animals reared in the *Montanera* system and pH_45_ (Figure 2B) differences in the meat from animals reared in the intensive system on account of their age at slaughter, such differences could not be related to the fibre-type populations, as there were no significant differences between them on account of age (Table 2 and Table 3). Such discrepancies could relate to the Iberian breed itself—that is, the fact that Iberian is a rustic breed with different pH behaviours in comparison to other improved pig breeds. In any case, the pH values and the pH rate decline identified in the muscles of animals reared in the intensive system and in the *Montanera* system can be deemed normal [41].

On the matter of the approximate composition, the high IMF values in fresh loins from younger animals reared in the intensive system (Table 6), as well as the higher IMF values in fresh loins from animals under the intensive system compared to those from *Montanera* animals (Table 7), might be associated with the animal growth rate [42]. Few studies associated a greater IMF content to a higher type I fibre content [16,43], but this association was not observed in the current study. More in detail, the decrease of the IMF with the age of the animal in the intensive system could compromise the marbling after the curing process [44]—dry-cured loin—considered a quality index by consumers [45].

The fact that there were no differences in water losses on account of animal age at slaughter in animals reared in the *Montanera* system (Table 5) could be explained by the significant impact of IMF, which remained the same as the animals grew up on them [46]. This association would also explain the smaller cooking losses that were identified in the fresh loins of animals reared in the intensive systems and slaughtered at 8 and 10 months of age in comparison to the animals slaughtered at 12 months of age (Table 6). The higher cooking losses of fresh Iberian loins from the oldest animals could have an impact on the texture. In fact, an increase in both the shear force measured by the WBSF test and hardness was observed in the latter with respect to the loins from the younger animals, although these differences were only significant for the former. This may condition the acceptance of these for fresh consumption by consumers. The literature has previously reported that fibre-type populations and sizes have an impact on water losses, specifically fast-twitch glycolytic (IIB) [16], and that hypertrophy [47] would act in detriment of the WHC. However, this association was not observed in the current study.

The higher a* values in the meat from animals slaughtered at older ages in both production systems (Table 5 and Table 6), as well as in the meat from animals reared in the *Montanera* in comparison to those reared in the intensive (Table 7) system, could be explained by the similar behaviour of myoglobin, given the close association between both parameters [48], which has been specifically identified for the Iberian pig [7]. At the same time, the increase in redness and in the myoglobin content cannot be associated in our study to the type of muscle fibres, since no differences were found in them on account of age (Table 2 and Table 3). On the other hand, as the type I fibre size increased with animal age, although differences were only significant for animals reared in the *Montanera* system (Table 2), the myoglobin content increased (Table 5), as reported in previous studies [49,50,51]. Age-related colour differences are important, as one of the characteristics of meat and meat products that influences their overall quality is the deep, dark, reddish colour, which is achieved by the myoglobin content [52].

On the matter of textural properties, these are closely related to muscle fibres. Thus, Ryu and Kim [53] suggested that, as the fibre size increases, the tenderness decreases, and Renand, Picard, Touraille, Berge and Lepetit [54] indicated a positive correlation between the tenderness and type I fibre population. This behaviour could explain, at least partially, the differences identified in the meat on account of the production system used for such animals. Specifically, a lower fibre type I population and larger size of type II fibres in LT muscles from animals reared in intensive systems (Table 4) would give rise to a higher hardness of their meats (Table 7). However, the findings of this study suggest the impact of other factors on top of the muscle fibre characteristics on the texture of meat. In fact, although the size of all types of fibres in the LT muscles proved to increase with animal age for the animals reared in the *Montanera* system, the texture characteristics remained unchanged. This could be explained by other production factors, such as exercise and IMF [55], the values being the same for the three animal batches. On the other hand, a lower IMF content in the fresh loins from animals reared in the intensive system and slaughtered at 12 months old could be responsible for a higher WBSF [56].

## 5. Conclusions

This study is an attempt to shedd light on Iberian fresh meat quality traits and muscle fibre characteristics as affected by animal age at slaughter and production system. Our findings suggest that the fibre-type population of *Longissimus thoracis* muscles from Iberian × Duroc crossed pigs would not be affected by animal age at slaughter. However, differences in the size of the fibres of the *Longissimus thoracis* muscles were observed on account of the animal age at slaughter reared under the *Montanera* system.

In terms of meat quality traits, variations in some quality parameters in the fresh loins from animals under both the *Montanera* and intensive conditions due to animal slaughter age were found, thus proving the latter to be a factor of variability of the Iberian fresh meat quality. A redder meat, due to a higher content of the pigment myoglobin, could be obtained with the age of the animal regardless of the production system. On the other hand, the increase in age would lead to less fat infiltration, greater losses during cooking and, also, to greater toughness in the case of meats from animals under the intensive system, and therefore, the most tender meat was obtained from animals slaughtered at a younger age (8 and 10 months).

Future studies should evaluate whether the differences observed in the fibrillar structures and quality parameters due to the age at slaughter and production system of the animals are perceived at the sensory level, as well as their impact on the technological curing process.

## Figures and Tables

**Table 1:** Characterization of the commercial feedstuffs and acorn and grass provided to animals under both the *Montanera* and intensive production systems.

	*Montanera* System			Intensive System	
	Commercial Feedstuffs	Acorn ^1^ (Kernel)	Grass ^1^	Commercial Feedstuffs	
Live weight (kg)	From 71.3 ± 1.64 to 102.8 ± 0.48	From 102.8 ± 0.48 to slaughtering		From 77.2 ± 2.93 to 111.5 ± 2.64	From 111.5 ± 2.64 to slaughtering
DM (%)	91.47 ± 0.10	56.5 ± 0.51	13.8 ± 0.11	91.1 ± 0.31	91.3 ± 0.47
Crude protein (%)	16.42 ± 0.20	6.5 ± 0.43	4.0 ± 1.40	15.7 ± 0.74	14.8 ± 0.91
Crude fat (%)	4.31 ± 0.10	7.2 ± 0.21	0.5 ± 0.03	3.0 ± 0.58	3.4 ±0.96
Crude fibre (%)	5.85 ± 0.07	6.6 ± 0.15	18.8 ± 0.92	5.5 ± 0.50	3.5 ± 0.53
Ash (%)	5.3 ± 0.03	2.10 ± 0.11	12.6 ± 0.50	5.4 ± 0.25	6.0 ± 0.21
NFE (%)	68.0 ± 0.20	77.7 ± 1.90	28.6 ± 1.60	58.5 ± 0.94	60.2 ± 1.53

Data are expressed on as the arithmetic mean ± standard error for dry matter. DM, dry matter; NFE, nitrogen-free extract. ^1^ Calculated from the average of three samplings: at the beginning, middle and end of the free-range finishing phase in *Montanera* (6 November 2018 to 12 January 2019).

**Table 2:** Muscle fibre population and size of m. *Longissimus thoracis* from Iberian × Duroc crossed animals reared under the *Montanera* system according to slaughter age.

	*Montanera* System			
	12 Months	14 Months	16 Months	*p*-Value
fibre type population (%)				
Type I	22.42 ± 2.42	24.67 ± 3.07	25.41 ± 3.64	0.813
Type IIA	19.20 ± 0.42	21.76 ± 0.98	19.68 ± 0.58	0.080
Type IIB	40.67 ± 1.20	35.36 ± 4.22	30.94 ± 2.17	0.126
Type IIX	21.39 ± 1.47	20.41 ± 1.44	23.48 ± 2.58	0.584
minor diameter measurement (µm)				
Type I	82.28 ± 4.71 ^b^	89.65 ± 1.89 ^ab^	98.38 ± 1.36 ^a^	0.017
Type IIA	76.80 ± 1.89 ^b^	77.44 ± 3.49 ^b^	96.31 ± 1.59 ^a^	0.001
Type IIB	79.15 ± 1.36 ^b^	87.39 ± 2.77 ^b^	100.20 ± 3.41 ^a^	0.002
Type IIX	75.08 ± 2.18 ^b^	75.50 ± 2.23 ^b^	96.17 ± 3.52 ^a^	0.000

Effect: animal age at time of slaughter: 12, 14 and 16 months (8 m. *Longissimus thoracis* per age set). Data expressed as arithmetic mean ± standard error. Type I, slow-twitch oxidative; Type IIA, fast-twitch oxido-glycolytic; Type IIB, fast-twitch glycolytic and Type IIX, fast-twitch oxidative. Means within a row with different letters indicate differences due to animal slaughter age for *p* = 0.05, according to Tukey’s HSD test.

**Table 3:** Muscle fibre population and size of m. *Longissimus thoracis* from Iberian × Duroc crossed animals reared under the intensive system according to slaughter age.

	Intensive System			
	8 Months	10 Months	12 Months	*p*-Value
fibre type population (%)				
Type I	20.71 ± 1.59	18.44 ± 1.82	17.40 ± 0.36	0.420
Type IIA	19.43 ± 1.03	20.46 ± 0.62	21.43 ± 1.62	0.626
Type IIB	34.00 ± 0.89	38.70 ± 1.87	41.90 ± 2.03	0.065
Type IIX	21.42 ± 0.17	20.81 ± 0.65	20.02 ± 0.62	0.346
minor diameter measurement (µm)				
Type I	86.00 ± 3.08	95.41 ± 2.03	98.24 ± 2.89	0.066
Type IIA	79.55 ± 1.71	86.23 ± 2.82	84.58 ± 2.94	0.681
Type IIB	100.63 ± 4.08	107.71 ± 3.25	101.93 ± 1.96	0.434
Type IIX	85.82 ± 2.94	88.00 ± 2.76	87.49 ± 2.46	0.943

Effect: animal age at time of slaughter; 8, 10 and 12 months (8 m. *Longissimus thoracis* per age set). Data expressed as arithmetic mean ± standard error. Type I, slow-twitch oxidative; Type IIA, fast-twitch oxido-glycolytic; Type IIB, fast-twitch glycolytic and Type IIX, fast-twitch oxidative. *p* > 0.05 indicates no significant differences according to Tukey’s HSD test.

**Table 4:** Muscle fibre population and size of m. *Longissimus thoracis* from Iberian × Duroc crossed animals according to production system.

	Production System		
	*Montanera*	Intensive	*p*-Value
fibre type population (%)			
Type I	24.16 ± 2.71	18.85 ± 1.41	0.026
Type IIA	20.21 ± 0.80	20.44 ± 1.10	0.803
Type IIB	35.66 ± 3.11	38.20 ± 2.05	0.330
Type IIX	21.76 ± 1.84	20.75 ± 0.53	0.462
minor diameter measurement (µm)			
Type I	90.10 ± 3.95	93.22 ± 3.31	0.382
Type IIA	83.52 ± 4.62	83.46 ± 4.23	0.988
Type IIB	88.91 ± 4.55	103.42 ± 3.19	0.010
Type IIX	82.25 ± 4.85	87.10 ± 3.51	0.248

Effect: animal production system: *Montanera* vs. intensive (24 m. *Longissimus thoracis* per production system). Data expressed as arithmetic mean ± standard error. Type I, slow-twitch oxidative; Type IIA, fast-twitch oxido-glycolytic; Type IIB, fast-twitch glycolytic and Type IIX, fast-twitch oxidative. *p* ≤ 0.05 indicates significant differences according to Tukey´s HSD test.

**Table 5:** Quality traits measured in fresh loins (m. *Longissimus thoracis*) from Iberian × Duroc crossed animals reared under the *Montanera* system according to slaughter age.

*Montanera* System				
	12 Months	14 Months	16 Months	*p*-Value
Approximate composition				
DM ^a^	30.22 ^b^ ± 0.30	28.30 ^b^ ± 0.25	32.75 ^a^ ± 0.74	0.000
IMF	3.98 ± 0.24	3.65 ± 0.15	4.11 ± 0.35	0.870
Mgb ^b^	1.81 ^b^ ± 0.08	2.00 ^b^ ± 0.15	2.44 ^a^ ± 0.02	0.000
Water losses				
WHC ^c^	29.06 ± 0.49	30.80 ± 0.49	29.59 ± 0.22	0.054
Cook loss ^c^	20.53 ± 1.62	22.78 ± 1.17	22.74 ± 0.66	0.419
Instrumental colour				
L*	47.11 ± 0.21	46.97 ± 1.07	46.84 ± 0.81	0.975
a*	12.24 ^b^ ± 0.38	14.55 ^a^ ± 0.47	14.19 ^a^ ± 0.63	0.025
b*	8.13 ± 0.27	8.02 ± 0.36	7.92 ± 0.41	0.925
Textural properties				
WBSF (N/cm^2^)	72.84 ± 5.20	67.55 ± 8.24	69.29 ± 4.97	0.861
Hardness (N/cm^2^)	1.54 ± 0.09	1.84 ± 0.09	1.99 ± 0.13	0.068
Springiness (cm)	0.84 ± 0.02	0.85 ± 0.02	0.82 ± 0.02	0.553
Cohesivess	0.72 ± 0.01	0.72 ± 0.01	0.72 ± 0.01	0.737
Chewiness (N cm s^2^)	0.98 ± 0.05	1.04 ± 0.07	0.94 ± 0.11	0.727
Resilience	0.48 ± 0.01	0.46 ± 0.01	0.47 ± 0.02	0.415

Effect: animal age at time of slaughter: 12, 14 and 16 months (8 fresh loins per age set). Data expressed as arithmetic mean ± standard error. DM, dry matter; IMF, intramuscular fat; L*, lightness; a*, redness; b*, yellowness (instrumental colour coordinates are dimensionless, measured in the CIELab space); Mgb, myoglobin; WHC, water-holding capacity and WBSF, Warner-Braztler shear force test. ^a^ Expressed in grams per 100 g of muscle. ^b^ Expressed in mg per gram of muscle. ^c^ Expressed as g of water released per 100 g of muscle. Means within a row with different letters indicate differences due to animal slaughter age for *p* = 0.05, according to Tukey’s HSD test.

**Table 6:** Quality traits measured in fresh loins (m. *Longissimus thoracis*) from Iberian × Duroc crossed animals reared under the intensive system according to slaughter age.

Intensive System				
	8 Months	10 Months	12 Months	*p*-Value
Approximate composition				
DM ^a^	31.58 ± 0.27	31.39 ± 1.27	29.78 ± 1.64	0.436
IMF	6.87 ^a^ ± 0.69	6.65 ^a^ ± 0.52	4.65 ^b^ ± 0.99	0.048
Mgb ^b^	1.47 ^b^ ± 0.10	1.87 ^ab^ ± 0.07	1.98 ^a^ ± 0.33	0.033
Water losses				
WHC ^c^	27.66 ± 0.76	29.69 ± 0.90	29.64 ± 2.16	0.450
Cook loss ^c^	18.70 ^c^ ± 0.26	20.66 ^b^ ± 0.73	23.63 ^a^ ± 1.30	0.002
Instrumental colour				
L*	51.15 ± 0.35	51.87 ± 0.46	49.12 ± 2.36	0.201
a*	9.32 ^c^ ± 0.36	10.58 ^b^ ± 0.62	12.25 ^a^ ± 1.26	0.028
b*	7.77 ± 0.31	6.99 ± 0.49	7.18 ± 0.84	0.504
Textural properties				
WBSF (N/cm^2^)	43.24 ^b^ ± 3.29	45.19 ^b^ ± 3.39	76.40 ^a^ ± 4.54	0.014
Hardness (N/cm^2^)	2.34 ± 0.04	2.42 ± 0.35	2.58 ± 0.05	0.805
Springiness (cm)	0.84 ± 0.02	0.85 ± 0.02	0.86 ± 0.04	0.909
Cohesivess	0.73 ± 0.01	0.72 ± 0.01	0.72 ± 0.01	0.890
Chewiness (N cm s^2^)	1.51 ± 0.21	1.49 ± 0.21	1.60 ± 0.14	0.937
Resilience	0.48 ± 0.01	0.48 ± 0.01	0.48 ± 0.01	0.435

Effect: animal age at time of slaughter: 8, 10 and 12 months (8 fresh loins per age set). Data expressed as arithmetic mean ± standard error. DM, dry matter; IMF, intramuscular fat; Mgb, myoglobin; WHC, water-holding capacity; L*, lightness; a*, redness; b*, yellowness (instrumental colour coordinates are dimensionless, measured in the CIELab space) and WBSF, Warner-Braztler shear force test. ^a^ Expressed in grams per 100 g of muscle. ^b^ Expressed in mg per gram of muscle. ^c^ Expressed as g of water released per 100 g of muscle. Means within a row with different letters indicate differences due to animal slaughter age for *p* = 0.05, according to Tukey’s HSD test.

**Table 7:** Quality traits measured in fresh loins (m. *Longissimus thoracis*) from Iberian × Duroc crossed animals according to the production system.

Production System			
	*Montanera*	Intensive	*p*-Value
Approximate composition			
DM ^a^	30.42 ± 0.89	30.92 ± 2.06	0.553
IMF	3.92 ± 0.23	6.04 ± 1.74	0.000
Mgb ^b^	2.08 ± 0.12	1.77 ± 0.31	0.014
Water losses			
WHC ^c^	29.82 ± 0.49	28.99 ± 2.44	0.261
Cook loss ^c^	22.02 ± 1.21	21.00 ± 2.43	0.345
Instrumental colour			
L*	46.97 ± 0.73	51.05 ± 2.59	0.000
a*	13.66 ± 0.64	10.72 ± 1.69	0.000
b*	8.02 ± 0.33	7.31 ± 0.92	0.043
Textural properties			
WBSF (N/cm^2^)	69.89 ± 5.88	54.94 ± 8.27	0.035
Hardness (N/cm^2^)	1.79 ± 0.13	2.45 ± 0.19	0.000
Springiness (cm)	0.84 ± 0.02	0.85 ± 0.03	0.678
Cohesivess	0.72 ± 0.01	0.72 ± 0.01	0.826
Chewiness (N cm s^2^)	0.99 ± 0.07	1.53 ± 0.18	0.000
Resilience	0.47 ± 0.03	0.48 ± 0.01	0.322

Effect: animal production system: *Montanera* vs. intensive (24 fresh loins per production system). Data expressed as arithmetic mean ± standard error. DM, dry matter; IMF, intramuscular fat; Mgb, myoglobin; WHC, water-holding capacity; L*, lightness; a*, redness; b*, yellowness (instrumental colour coordinates are dimensionless, measured in the CIELab space) and WBSF, Warner-Braztler shear force test. ^a^ Expressed in grams per 100 g of muscle. ^b^ Expressed in mg per gram of muscle. ^c^ Expressed as g of water released per 100 g of muscle. *p* ≤ 0.05 indicates significative differences, according to Tukey´s HSD test.

## Data Availability

Data sharing not applicable.
